# Variant analysis of 1,040 SARS-CoV-2 genomes

**DOI:** 10.1371/journal.pone.0241535

**Published:** 2020-11-05

**Authors:** Eric C. Rouchka, Julia H. Chariker, Donghoon Chung

**Affiliations:** 1 Department of Computer Science and Engineering, University of Louisville, Louisville, Kentucky, United States of America; 2 Kentucky Biomedical Research Infrastructure Network Bioinformatics Core, University of Louisville, Louisville, Kentucky, United States of America; 3 Neuroscience Training Program, University of Louisville, Louisville, Kentucky, United States of America; 4 Department of Microbiology and Immunology, University of Louisville, Louisville, Kentucky, United States of America; John Curtin School of Medical Research, AUSTRALIA

## Abstract

The severe acute respiratory syndrome-coronavirus 2 (SARS-CoV-2) viral genome is an RNA virus consisting of approximately 30,000 bases. As part of testing efforts, whole genome sequencing of human isolates has resulted in over 1,600 complete genomes publicly available from GenBank. We have performed a comparative analysis of the sequences, in order to detect common mutations within the population. Analysis of variants occurring within the assembled genomes yields 417 variants occurring in at least 1% of the completed genomes, including 229 within the 5’ untranslated region (UTR), 152 within the 3’UTR, 2 within intergenic regions and 34 within coding sequences.

## Introduction

SARS-CoV-2, formerly known as the “Wuhan seafood market pneumonia virus,” is a novel coronavirus that first appeared at the seafood and wildlife wholesale market in Wuhan, Hubei Provence, China during late November/early December, 2019 [1]. Due to its high human-to-human transmission rate [2], longer than normal latent period [3] and mortality rates in vulnerable populations, a global pandemic was declared by the World Health Organization (WHO) on March 11, 2020 for the associated COVID-19 disease [4]. As of May 10, 2020, a total of 4,006,257 cases resulting in 278,892 deaths in 215 countries have been confirmed [5].

**Fig 1:**
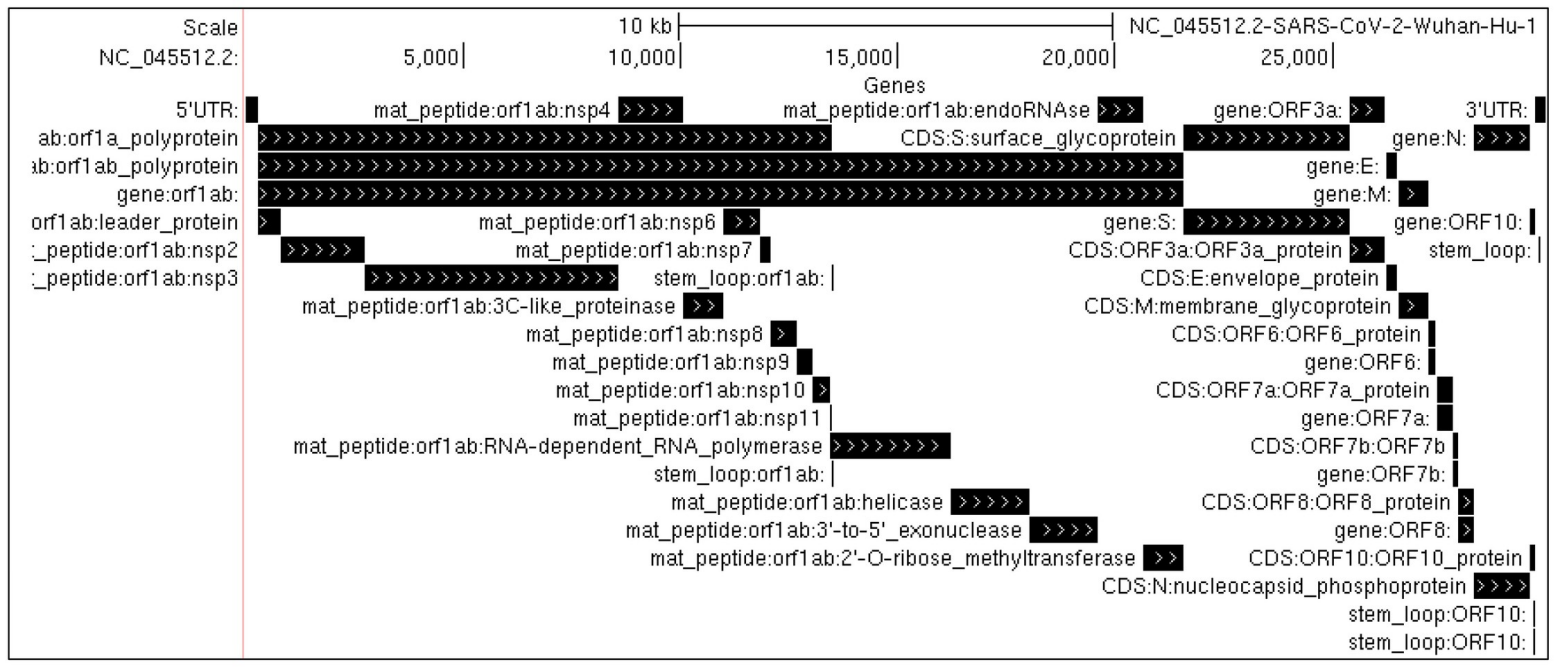
Genome structure of SARS-CoV-2. Shown are the locations of the 10 major open reading frames, as well as specific peptides and structural elements produced within them.

The reference genome of the SARS-Cov-2 RNA virus (GenBank accession NC_045512) consists of 29,903 bases. Among its features are a 265 base 5’ untranslated region (UTR) and a 3’ UTR composed of 229 bases. Its coding regions consist of 10 open reading frames (ORFs) coding for 26 genes [3], including 13,218 bases coding for the ORF1ab polyproteins, whose transcription includes a 1 bp ribosomal slippage event [6]; 3,822 bases coding for a spike surface glycoprotein (S), 228 bases coding a small envelope protein (E), 669 bases coding for a membrane glycoprotein protein (M) and 1,260 bases coding for a nucleocapsid protein (N) along with five additional ORFs (ORF3a, ORF6, ORF7a/ORF7b, ORF8, and ORF10) (Fig 1).

**Fig 2:**
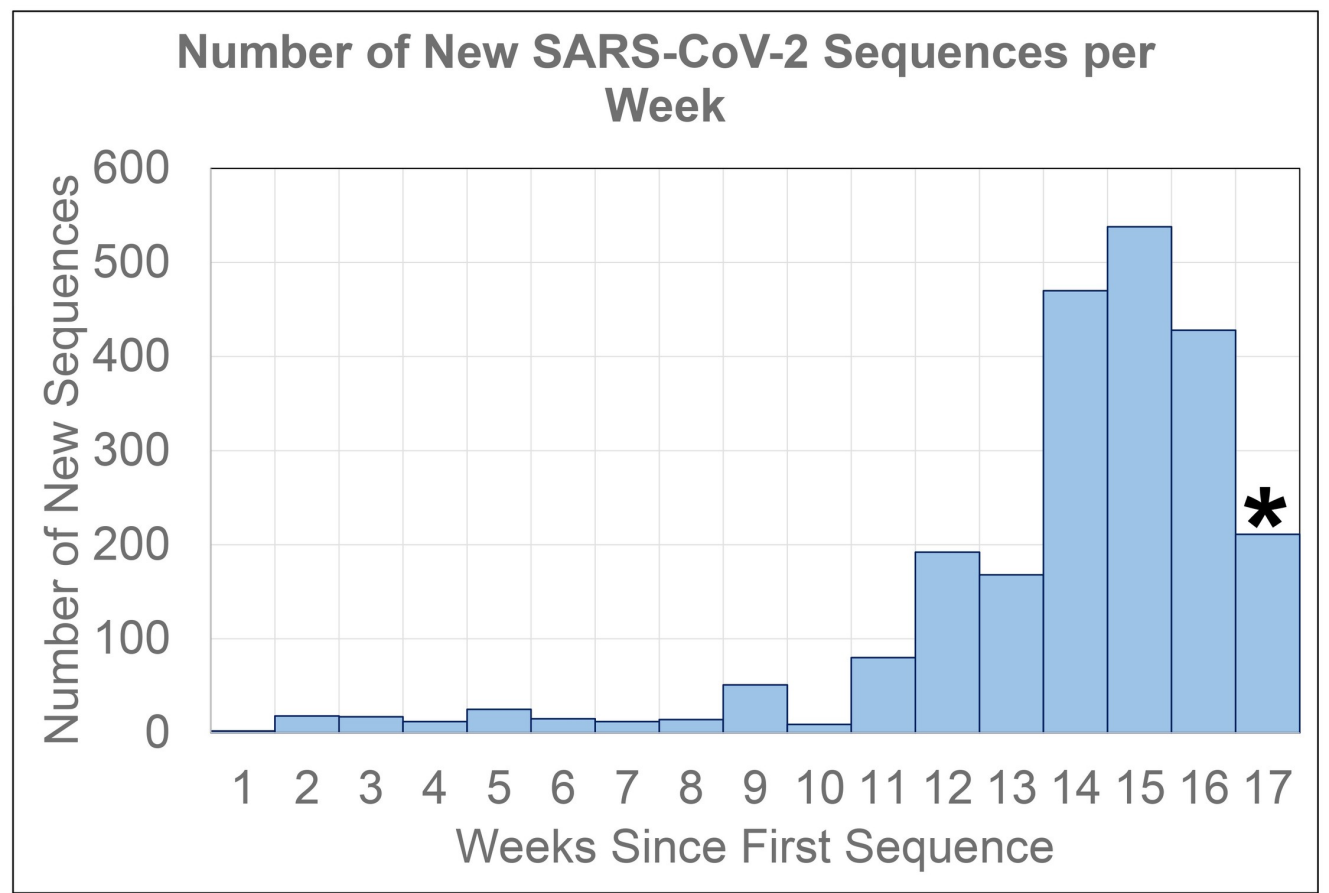
Number of new SARS-CoV-2 sequences deposited in GenBank on a weekly basis through May 7, 2020. *Week 17 is only a partial week.

Since the public release of the first reference sequence (MN908947; NC_045512) from NCBI’s GenBank [7] on January 12, 2020, the number of sequences available has increased exponentially, at a current rate of approximately 70 new sequences per day (Fig 2). Isolates have been sequenced from 27 countries (Table 1), as well as 34 states, Washington DC, and passengers from a cruise (Table 2).

**Table 1:** Isolate groups and geographic locations with identical SARS-CoV-2 genomic sequences.

Name	Num Seqs	Location	Other Locations
MULTIPLE_31	18	USA-WA	--
MULTIPLE_25	16	USA-WA	USA-NY,USA-NC,USA-UT
MULTIPLE_9	14	CHN-Zhejiang	--
MULTIPLE_8	13	USA-MA	USA-KS,USA-NC,USA-PA
MULTIPLE_21	12	USA-NY	--
MULTIPLE_11	9	USA-CruiseA	USA-IL
MULTIPLE_16	7	USA-GA	USA-SC
MULTIPLE_26	7	USA-NY	--
MULTIPLE_35	5	USA-NY	--
MULTIPLE_6	4	USA-MA	--
MULTIPLE_12	4	USA-IA	--
MULTIPLE_45	4	USA-NY	USA-RI,USA-OH
MULTIPLE_2	3	USA-MI	--
MULTIPLE_3	3	USA-NY	--
MULTIPLE_4	3	HKG	--
MULTIPLE_10	3	KOR	--
MULTIPLE_18	3	USA-NY	--
MULTIPLE_23	3	USA-NY	USA-NJ
MULTIPLE_24	3	USA-OH	USA-PA
MULTIPLE_28	3	CZE	--
MULTIPLE_29	3	HKG	--
MULTIPLE_33	3	USA-NH	--
MULTIPLE_37	3	USA-NY	--
MULTIPLE_40	3	USA-WA	--
MULTIPLE_43	3	CHN-Beijing	--
MULTIPLE_1	2	USA	USA-CA,USA-WA
MULTIPLE_5	2	USA-NY	--
MULTIPLE_7	2	CHN	--
MULTIPLE_13	2	USA-CA	--
MULTIPLE_14	2	--	USA-CA,TWN
MULTIPLE_15	2	USA-IN	--
MULTIPLE_17	2	USA-IA	--
MULTIPLE_19	2	USA-WA	--
MULTIPLE_20	2	CHN	--
MULTIPLE_22	2	TWN	--
MULTIPLE_27	2	USA-NY	--
MULTIPLE_30	2	USA-NY	--
MULTIPLE_32	2	USA-FL	--
MULTIPLE_34	2	ESP	--
MULTIPLE_36	2	USA-NE	--
MULTIPLE_38	2	USA-CA	--
MULTIPLE_39	2	USA-MI	--
MULTIPLE_41	2	USA-WA	--
MULTIPLE_42	2	USA	--
MULTIPLE_44	2	USA-CA	--
MULTIPLE_46	2	--	USA-PA,USA-VA
MULTIPLE_47	2	USA-NY	--

**Table 2:** Number of SARS-CoV-2 isolate sequences extracted from GenBank by country.

Country	Count	Country	Count
USA	1025	MYS	2
CHN	64	PAK	2
TWN	21	VNM	2
HKG	19	AUS	1
IND	10	FRA	1
ESP	7	IRN	1
PRI	5	ITA	1
GRC	4	NPL	1
KAZ	4	PER	1
KOR	4	SWE	1
LKA	4	TUR	1
BRA	2	ZAF	1
ISR	2		

SARS-CoV-2 is postulated to have originated from zoonotic transfer of a pangolin betacoronavirus based on a phylogenetic analysis of coronavirus sequences, due to a common insertion of 12 nucleotides within the receptor binding domain of the S protein region that optimizes binding to the human ACE2 receptor, although the most similar betacoronavirus is the bat RaTG13 [8]. RNA viruses are characterized by a high mutation rate [9] driven by RNA dependent RNA polymerase (RdRp) that results in viral evolution [10]. Given the importance of the genome sequence with host transmission (in particular the S protein), it is important to understand common mutations within the population in order to have a better handle on viral load, virus spread, virus evolution, and disease severity.

A number of previous studies have examined variants within SARS-CoV-2 isolates. One prior study looking at the genome diversity of SARS-CoV-2 have identified 93 mutations occurring in at least one isolate from a set of 86 complete genomes [11]. A second report from a set of 220 complete genomes identified eight novel recurring mutations, with specific prevalence within Asian, North American, and European populations [10]. This study by Pachetti, et. al, also showed the occurrence of mutations over time based on sequence sampling dates. A report by Yin [12] identified fifteen high-frequency single nucleotide polymorphisms when comparing a set of 558 SARS-CoV-2 strains. This study found four of these mutations, 241C > T, 3037C > T, 14408C > T, and 23403A > G to be more prevalent in European strains, where the COVID-19 is typically more severe. Wang et. al [13] detected 13 variation sites among 95 full-length genomic sequences (variants occurring in at least 3 isolates), with two at positions 8,782 and 28,144 showing a high mutation rate around 30%. Khailany et. al [14] looked at mutations within 95 complete genome sequences, and found 116 mutations occurring in at least one isolate, with the most common being 8762C > T, 28144T > C, and 29095C > T. Tang et. al [15] studied mutations across 103 strains, and determined there were mutations in 149 sites, including six nonsynonymous mutations occurring at least twice. Additionally, this study identified two different mutations (with near complete linkage between 8782T > C and 28144C > T) that separated the virus into two groups labeled L and S. Using deep sequencing reads, Tang et. al also identified 18 locations showing intrahost variants, demonstrating heterogeneity of the virus within a specific host. Wang et. al [16] identified ten high frequency mutations within a set of 108 isolates, which they say can be used to classify SARS-CoV-2 into five main groups. The mutations published include 28144T > C,8782C > T, 23403A > G, 3037C > T, 11083G > T, 26144G > T, 2261G > T.

Forster et. al [17] used phylogenetic network analysis to find three central variants, A, B, and C, distinguishing East Asian isolates from European and American isolates. In their study, variants found defined clusters and/or subclusters by synonymous mutations 29095T > C (subclustering A), synonymous mutation 8782T > C and nonsynonymous mutation 28144C > T (separation of clusters A and B). In addition, Shen et. al [18] observed a median of 1–4 intrahost variants, ranging from 0–51, from a set of eight patients infected with SARS-CoV-2. Included in the Shen study were two patients from the same household, one of which was likely to have infected the other. Interestingly, only 7/25 variants detected in these two individuals were shared, illustrating the high viral mutation rate.

The SARS-CoV-2 spike (S) protein is of particular interest, due to its interaction with the human ACE2 protein that helps to mediate infection of host cells. Korber, et. al [19] have set up a workflow for measuring the dynamics of nonsynonymous mutations within the S protein coding region. This study has uncovered a number of amino acid mutations in the S protein, including D614G (23403G > A), which is thought to increase transmissibility due to its rapid expansion in global samples. This mutation was shown to have a high association with two other mutations, 3037C > T and 14409C > T.

## Materials and methods

All available SARS-CoV-2 nucleotide sequences and associated annotations (including locality) were downloaded from NCBI (https://www.ncbi.nlm.nih.gov/genbank/sars-cov-2-seqs/) on 5/7/2020, resulting in 2,262 sequences. Geographical location information was reduced to the corresponding ISO 3166–1 alpha-3 country code (S1 Table), and the two-letter abbreviated state code. Sequences were then filtered to include only those listed as complete with an isolation source of “Homo sapiens”, which left 1,695 sequences listed as complete genomes isolated from human samples. A total of 538 sequences from this set containing gaps in their assembly (defined by the character “N”) were removed from further analysis, leaving 1,157 complete, ungapped genomes. One-hundred and fifty-nine (159) isolate sequences were represented two or more times, resulting in 45 unique genomes from the set of 159. The isolates that were 100% matches across their entire length were merged into a single representative sequence (Table 3). A final total of 1,043 unique complete genomic sequences without gaps were used in the final analysis, including three from Kentucky that we previously analyzed [20]. These 1,043 sequences were then compared against each other, and a distance matrix was constructed based on the number of gaps and mismatches, as calculated by NCBI blastn (v2.10.0) [21]. A multiple sequence alignment was performed using kalign (v3.2.5) [22] which resulted in a.aln alignment file which was used for further detection and analysis of variants. The results from kalign were used as input into a custom perl script, findVariants.pl, which determined all minor allele frequencies, their genomic positions, overlap with gene annotations, and effect on codons and amino acids. The alignment file as well as other supporting data and scripts are available on our github site: https://github.com/UofLBioinformatics/SARS_CoV_2_Variants.

**Table 3:** Number of SARS-CoV-2 isolate sequences extracted from GenBank by state/region.

State/Region	Count	State/Region	Count
USA-WA	334	USA-SC	7
USA-NY	185	USA-AZ	6
USA-CA	115	USA-TX	5
USA-MI	44	USA-IN	4
USA-VA	37	USA-LA	4
USA-CT	28	USA-NH	4
USA-CruiseA	25	USA-OR	4
USA-ID	22	USA-RI	4
USA-MA	19	USA-KY	3
USA-GA	15	USA-NJ	3
USA-FL	10	USA-OH	3
USA-UT	10	USA-HI	2
USA-WI	10	USA-MD	2
USA-PA	9	USA-NE	2
USA-IA	7	USA-NV	2
USA-IL	7	USA-DC	1
USA-MN	7	USA-KS	1
USA-NC	7	USA-MO	1

Variants were analyzed for their linkage using Haploview (v4.2) which generates a logarithm of odds (LOD) score to statistically describe whether two variants are likely to be inherited together based on their covariance and chromosomal position. In the case of a small genome, such as SARS-CoV-2, the covariance described by the r^2^ correlation coefficient is likely to provide the most informative information relating two variants.

## Results

**Fig 3:**
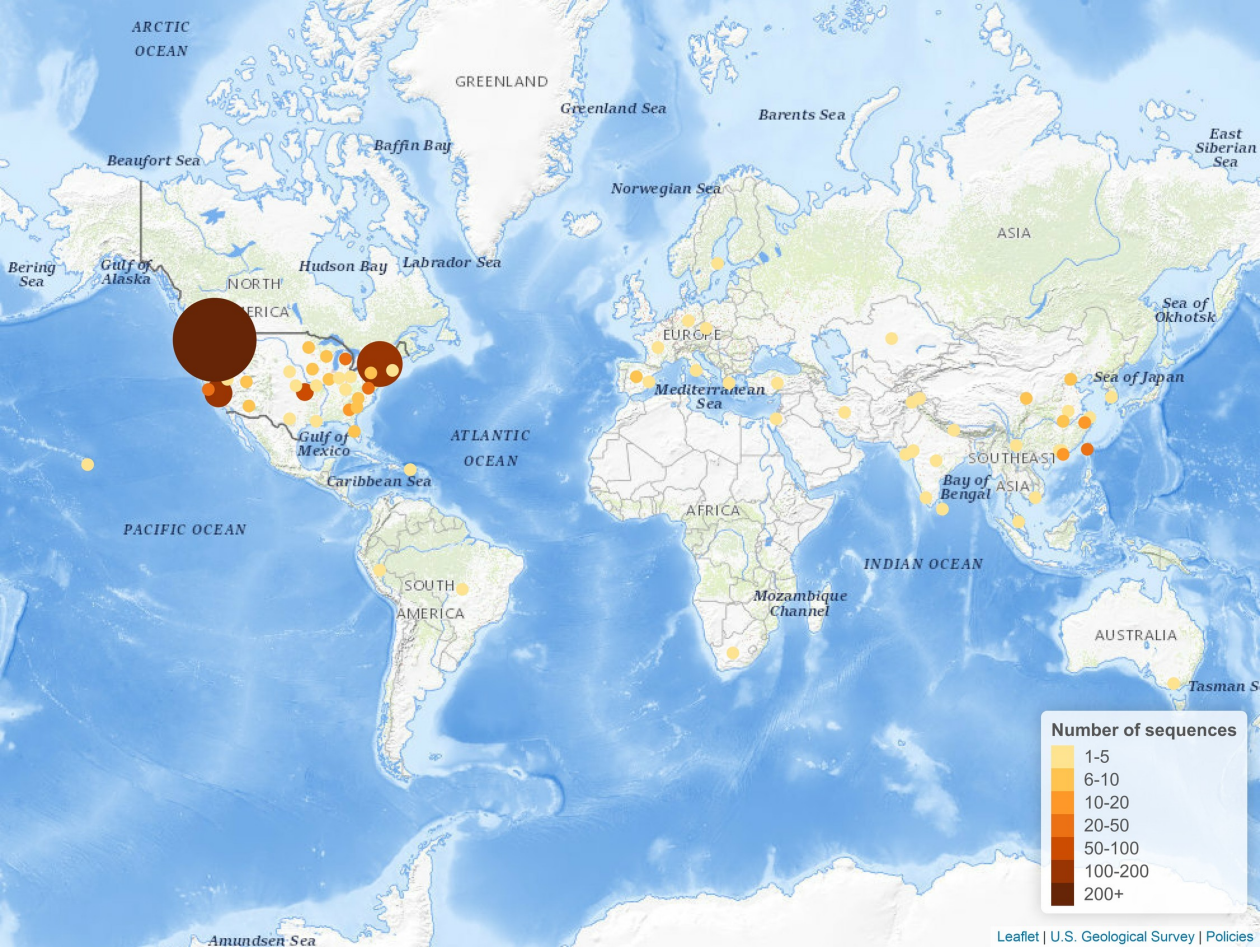
Distribution of the number of SARS-CoV-2 sequences in GenBank worldwide. Figure created using Leaflet (leafletjs.com) with tiles from The National Map. Map services and data available from U.S. Geological Survey, National Geospatial Program.

**Fig 4:**
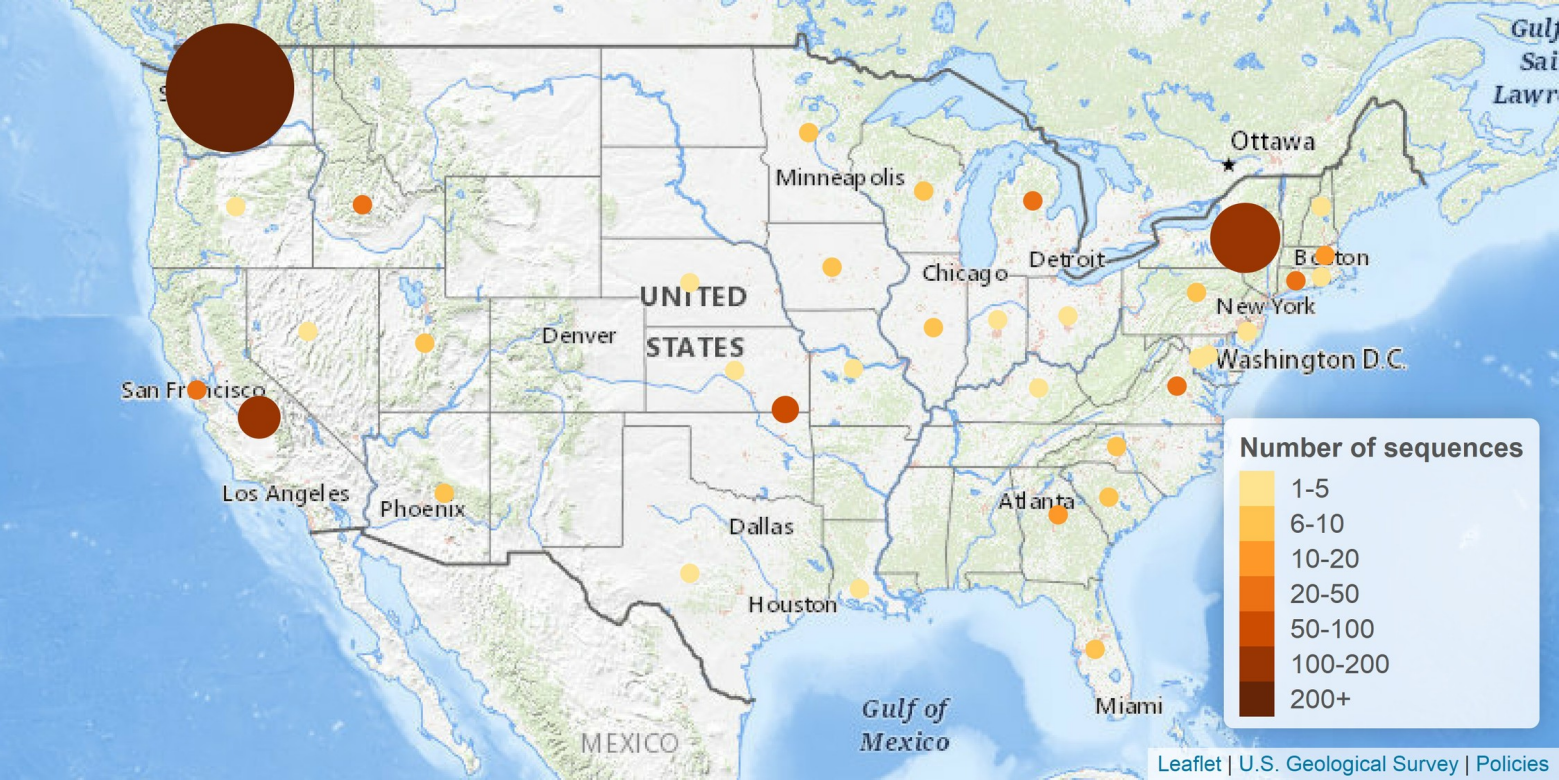
Distribution of the number of SARS-CoV-2 sequences in the United States available in GenBank. Figure created using Leaflet (leafletjs.com) with tiles from The National Map. Map services and data available from U.S. Geological Survey, National Geospatial Program.

Our analysis focused on a set of 1,043 filtered sequences, including 1,040 publicly available in GenBank, as well as three new isolates sequenced by our group in Kentucky (GenBank accessions MT365025, MT365026, and MT365027). From the non-filtered group of complete and ungapped SARS-CoV-2 genomic sequences, we found 47 groups of sequences that had at least two isolates that were 100% identical, including one group with 18 isolates all from Washington state; a second group with 16 isolates primarily from Washington state, and one group of 14 isolates all from Zhejiang, China (Table 1). These clustered groups of sequences are not surprising, particularly when examining the geographical similarities, suggesting clusters of similar transmission in both time and viral strains. The majority of the sequences were from the United States (Table 2, Fig 3), with California, New York, and Washington comprising the majority of the sequences (Table 3, Fig 4).

Based on a variant threshold of minor allele frequencies of 1% or more, we detected a total of 417 locations where isolate genomes express an alternate allele than the reference, NC_045512, including 229 in the 5’UTR, 21 in ORF1ab, 2 in S, 3 in ORF3a, 1 in M, 3 in ORF8, 4 in N, 2 intergenic, and 152 in the 3’UTR. The vast majority of the detected variants lie within the 5’UTR and 3’UTR, and represent deletion events. Since these may be due to a number of factors such as sequencing preparation (i.e. selection of amplicon primers) and difficulty in multiple sequence alignment construction that contribute to less reliability, we only retained seven of the UTR variants for further consideration. These seven UTR variants represent non-indel events which are unlikely to be sequencing artifacts. Overall, our filtering led to a total of 44 variants (Table 4). Of these, twenty have previously been reported (S2 Table), including the pair at locations 8782/28144 which was previously demonstrated to have a high linkage [15] and the tuples at locations 8782/18060/28144, 241/3037/23403/28144, and 241/3037/14408/23403 which were shown to have a high number of descendants [12]. We uncovered 24 novel variants not previously described to our knowledge, which may be a result of more recent mutation events and/or fixation of a mutation within the population. Surprisingly, two of these, 1059C > T in nsp2 region of ORF1ab and 25563G > T in ORF3a are found at a high frequency within the population, at a rate of 43% and 49%, respectively.

**Table 4:** Common variants (> 1%) detected within SARS-CoV-2 isolates.

Reference		Allele Counts		Nucleotide			Codon			Amino Acid		
Position	Region		MAF	Cons	Alt	Ref	Cons	Alt	Ref	Cons	Alt	Ref
50	5’UTR	C:811;-:220;T:10;A:2	0.010	C	T	C	NA	NA	NA	NA	NA	NA
241	5’UTR	T:599;C:436;-:7;Y:1	0.418	T	C	C	NA	NA	NA	NA	NA	NA
490	ORF1ab1	T:1022;A:20;W:1	0.192	T	A	T	GAT	GAA	GAT	Asp	Glu	Asp
833*	ORF1ab1	T:1011;C:32	0.031	T	C	T	TTC	CTC	TTC	Phe	Leu	Phe
1059	ORF1ab1	C:593;T:449;Y:1	0.430	C	T	C	ACC	ATC	ACC	Thr	Ile	Thr
1397	ORF1ab1	G:1030;A:13	0.013	G	A	G	GTA	ATA	GTA	Val	Ile	Val
2416	ORF1ab1	C:1023;T:20	0.089	C	T	C	TAC	TAT	TAC	Tyr	Tyr	Tyr
3037	ORF1ab1	T:586;C:457	0.438	T	C	C	TTT	TTC	TTC	Phe	Phe	Phe
3177	ORF1ab1	C:1022;T:20;Y:1	0.192	C	T	C	CCT	CTT	CCT	Pro	Leu	Pro
6040	ORF1ab1	C:1028;T:15	0.014	C	T	C	TTC	TTT	TTC	Phe	Phe	Phe
8782	ORF1ab1	C:791;T:251;Y:1	0.241	C	T	C	AGC	AGT	AGC	Ser	Ser	Ser
11083	ORF1ab1	G:973;T:69;C:1	0.066	G	T	G	TTG	TTT	TTG	Leu	Phe	Leu
11916*	ORF1ab1	C:1008;T:35	0.036	C	T	C	TCA	TTA	TCA	Ser	Leu	Ser
14408	ORF1ab2	T:603;C:440	0.422	T	C	C	CTT	CCT	CCT	Leu	Pro	Pro
14805	ORF1ab2	C:1003;T:40	0.038	C	T	C	TAC	TAT	TAC	Tyr	Tyr	Tyr
17247	ORF1ab2	T:1018;C:25	0.024	T	C	T	CGT	CGC	CGT	Arg	Arg	Arg
17747	ORF1ab2	C:850;T:189;Y:4	0.181	C	T	C	CCT	CTT	CCT	Pro	Leu	Pro
17858	ORF1ab2	A:851;G:192	0.184	A	G	A	TAT	TGT	TAT	Tyr	Cys	Tyr
18060	ORF1ab2	C:849;T:194	0.186	C	T	C	CTC	CTT	CTC	Leu	Leu	Leu
18736*	ORF1ab2	T:1024;C:19	0.018	T	C	T	TTT	CTT	TTT	Phe	Leu	Phe
18877	ORF1ab2	C:1004;T:39	0.037	C	T	C	CTA	TTA	CTA	Leu	Leu	Leu
18998*	ORF1ab2	C:1017;T:26	0.025	C	T	C	GCA	GTA	GCA	Ala	Val	Ala
20268	ORF1ab2	A:1031;G:12	0.012	A	G	A	TTA	TTG	TTA	Leu	Leu	Leu
23403	S	G:602;A:439;R:2	0.421	G	A	A	GGT	GAT	GAT	Gly	Asp	Asp
24034	S	C:1017;T:25;Y:1	0.024	C	T	C	AAC	AAT	AAC	Asn	Asn	Asn
25563*	ORF3a	G:536;T:507	0.486	G	T	G	CAG	CAT	CAG	Gln	His	Gln
25692	ORF3a	C:1022;T:21	0.020	C	T	C	GGC	GGT	GGC	Gly	Gly	Gly
26144	ORF3a	G:999;T:44	0.042	G	T	G	GGT	GTT	GGT	Gly	Val	Gly
26729	M	T:1020;C:22;Y:1	0.021	T	C	T	GCT	GCC	GCT	Ala	Ala	Ala
27964	ORF8	C:990;T:52;-:1	0.050	C	T	C	TCA	TTA	TCA	Ser	Leu	Ser
28077	ORF8	G:1017;C:22;T:2;-:1;S:1	0.021	G	C	G	GTG	CTG	GTG	Val	Leu	Val
28144	ORF8	T:791;C:250;-:1;Y:1	0.240	T	C	T	TTA	TCA	TTA	Leu	Ser	Leu
28688	N	T:1032;C:11	0.011	T	C	T	TTG	CTG	TTG	Leu	Leu	Leu
28881	N	G:990;A:53	0.051	G	A	G	AGG	AAG	AGG	Arg	Lys	Arg
28882	N	G:991;A:52	0.050	G	A	G	AGG	AGA	AGG	Arg	Arg	Arg
28883	N	G:991;C:52	0.050	G	C	G	GGA	CGA	GGA	Gly	Arg	Gly
29540	INTERGENIC	G:1017;A:26	0.025	G	A	G	NA	NA	NA	NA	NA	NA
29553	INTERGENIC	G:939;A:104	0.100	G	A	G	NA	NA	NA	NA	NA	NA
29700	3’UTR	A:1023;G:20	0.019	A	G	A	NA	NA	NA	NA	NA	NA
29742	3’UTR	G:1025;T:11;A:4;-:3	0.011	G	T	G	NA	NA	NA	NA	NA	NA
29864	3’UTR	G:746;-:275;A:19;T:1;R:1;V:1	0.018	G	A	G	NA	NA	NA	NA	NA	NA
29867	3’UTR	T:645;-:361;A:37	0.035	T	A	T	NA	NA	NA	NA	NA	NA
29868	3’UTR	G:625;-:367;A:39;C:8;R:3;D:1	0.051	G	A	G	NA	NA	NA	NA	NA	NA
29870	3’UTR	C:608;-:378;A:50;M:5;H:1;T:1	0.048	C	A	C	NA	NA	NA	NA	NA	NA

Cons: alignment consensus; Alt: alignment alternative; Ref: NC_045512 reference sequence. Note that the ORF1ab open reading frame is split into two parts, ORF1ab1 which occurs from bases 266…13468 in the reference sequence, and ORF1ab2 which occurs from bases 13468…21555 as result of a polymerase slippage event. MAF: Minor allele frequency

*Previously unreported nonsynonymous mutation.

One of the variants found, 29742G > T, occurs within the 3’ UTR stem loop II-like motif (s2m) which plays a role in viral replication and recruitment of host transcriptional machinery [23]. This mutation therefore may affect the folding of the s2m motif. None of the other non-coding mutations are found within known structural elements.

**Fig 5:**
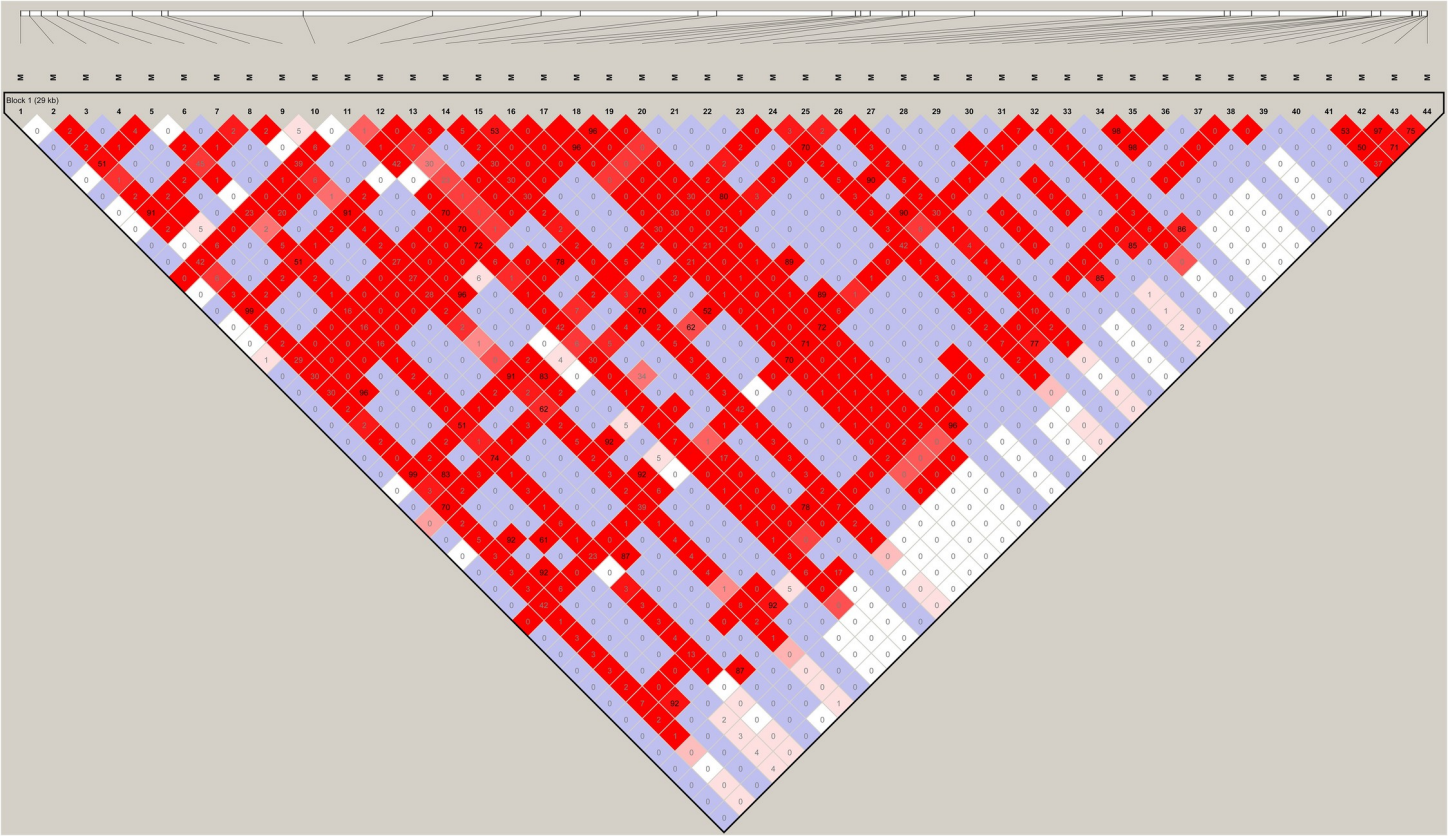
Linkage disequilibrium between SARS-CoV-2 variants. Shown is the Haploview pairwise association between variants. The colors are determined based on the default D’/LOD score. The value shown in each cell is the r^2^ value.

**Fig 6:**
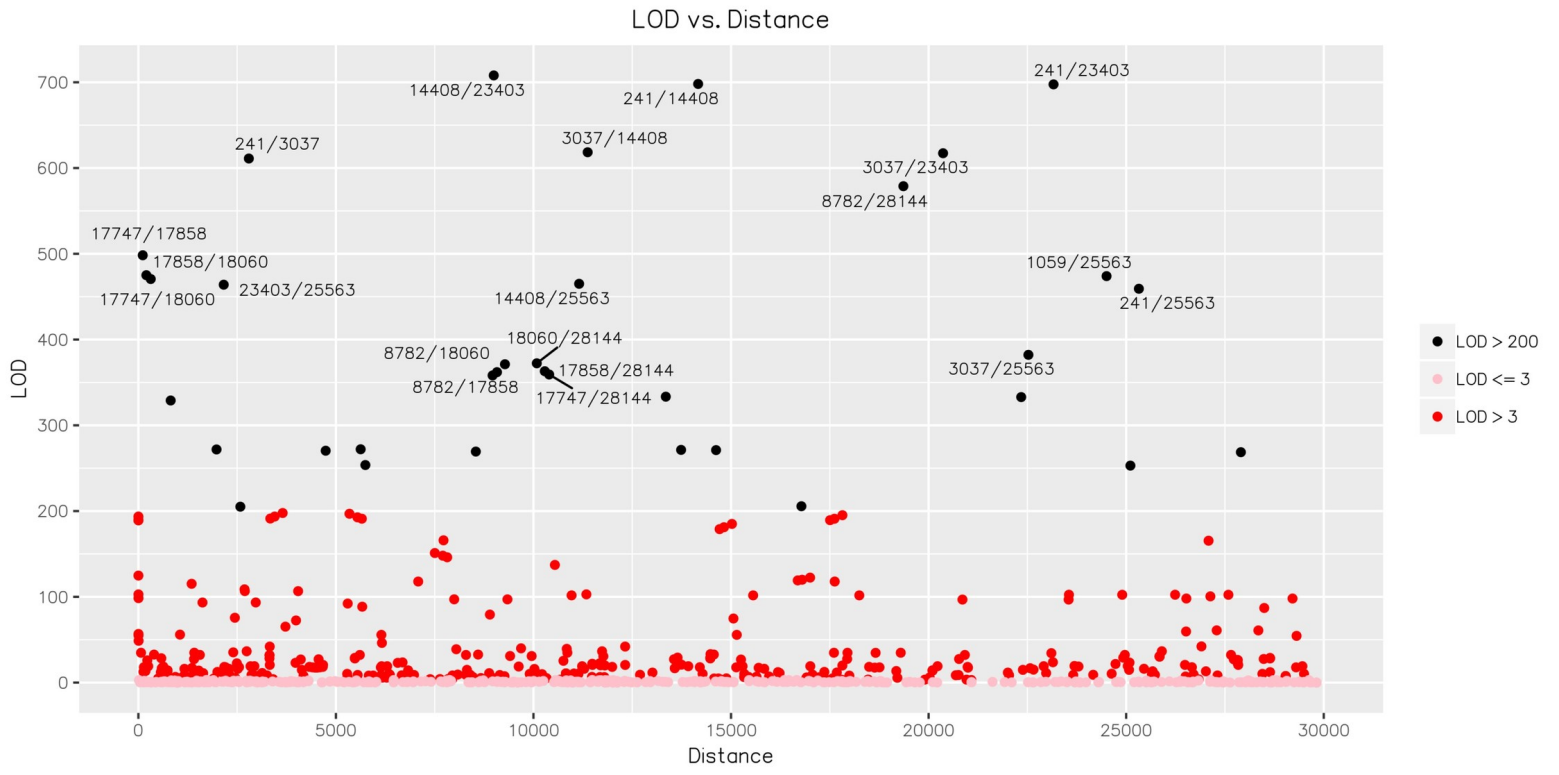
LOD of each variant pair vs. their distance. The points in black represent variant pairs with a LOD score > 200. The twenty pairs with the highest LOD scores are labeled based on their positions within the reference genome.

**Fig 7:**
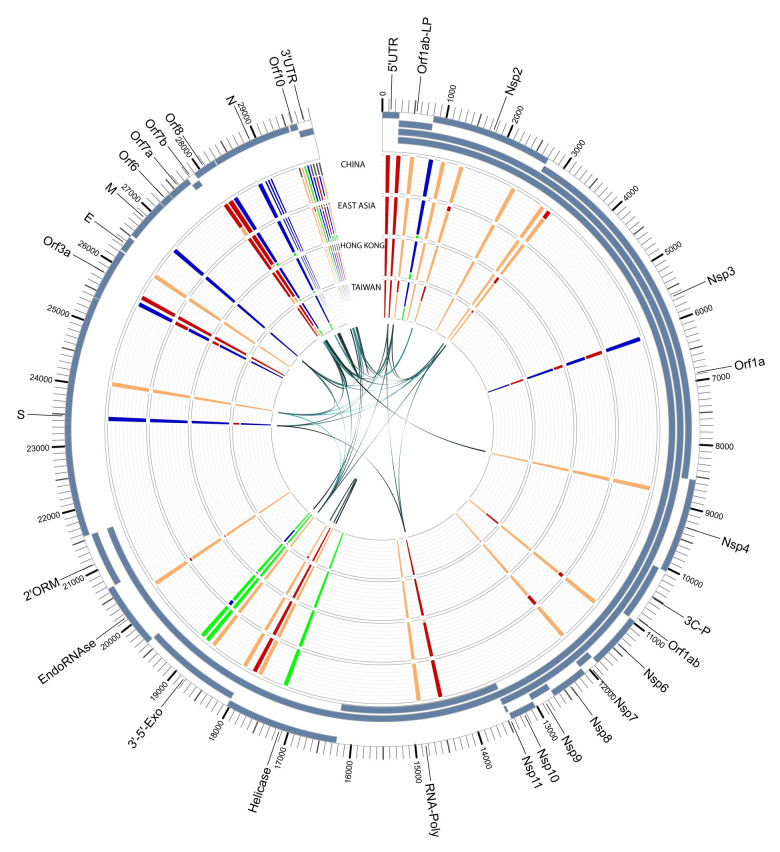
SARS-CoV-2 genome variants for China, East Asia, Hong Kong, and Taiwan. The outer track shows the gene/protein coding regions displayed in blue. The four inner tracks display nucleotide frequencies (green (A), orange (C), blue (G), and red (T)) at 44 locations with an alternate allele frequency > 1%. The inner-most track of arcs shows variant locations having a high linkage with r^2^ values ranging from 0.8 (narrow, light) to 1 (wide, dark). Abbreviations: leader protein (LP), 3C-like-proteinase (3C-P), RNA-dependent-RNA-polymerase (RNA-Poly), 3′-to-5′-exonuclease (3′-5′-Exo), 2′-O-ribose-methyltransferase (2′ORM).

**Fig 8:**
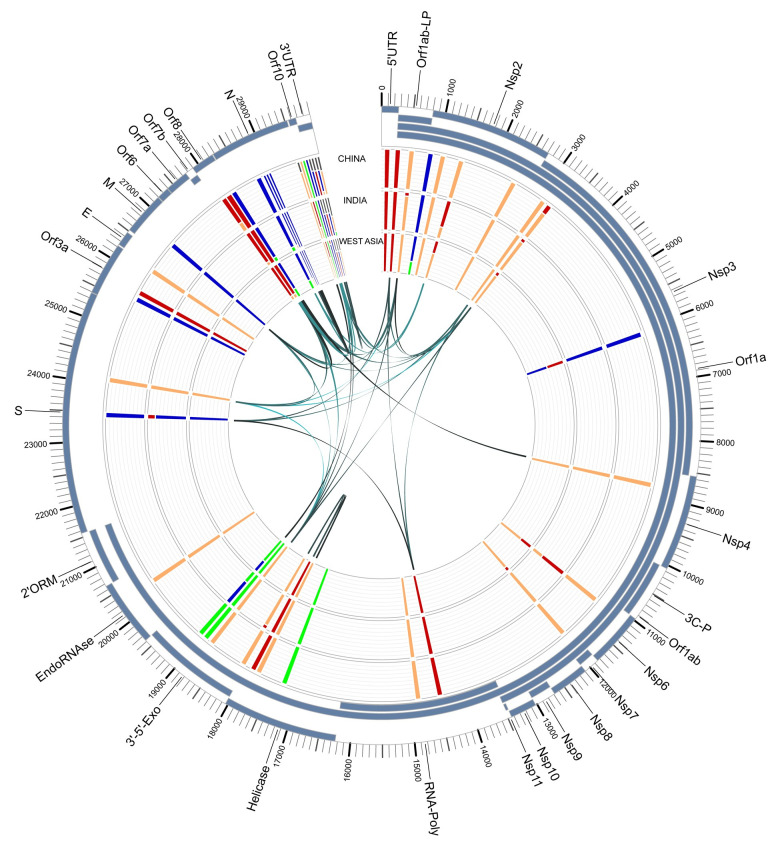
SARS-CoV-2 genome variants for China, India, and West Asia. The outer track shows the gene/protein coding regions displayed in blue. The four inner tracks display nucleotide frequencies (green (A), orange (C), blue (G), and red (T)) at 44 locations with an alternate allele frequency > 1%. The inner-most track of arcs shows variant locations having a high linkage with r^2^ values ranging from 0.8 (narrow, light) to 1 (wide, dark). Abbreviations: leader protein (LP), 3C-like-proteinase (3C-P), RNA-dependent-RNA-polymerase (RNA-Poly), 3'-to-5'-exonuclease (3’-5’-Exo), 2'-O-ribose-methyltransferase (2’ORM).

**Fig 9:**
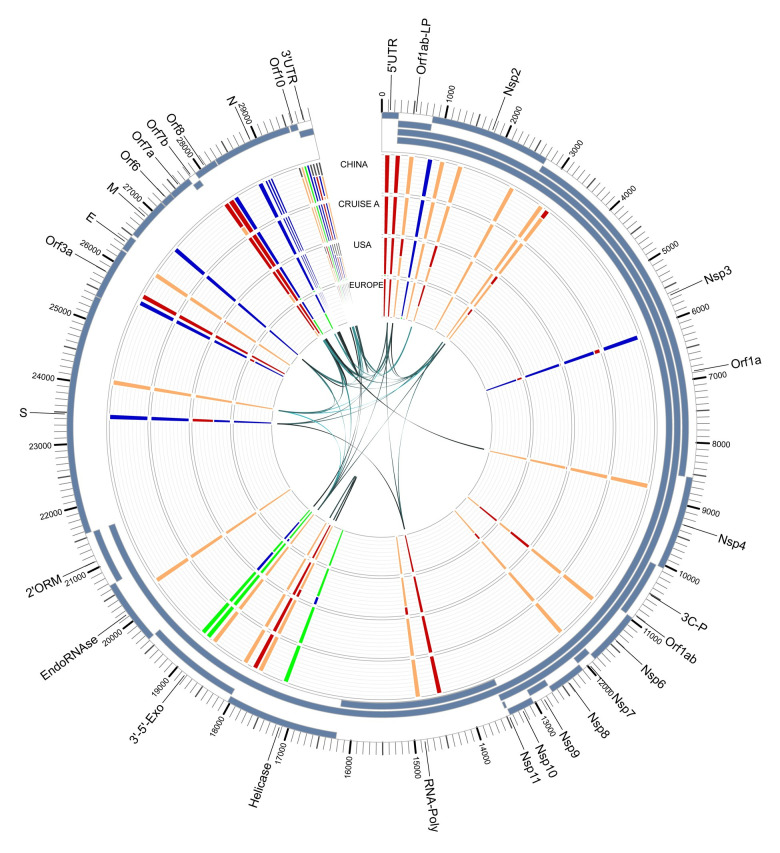
SARS-CoV-2 genome variants for China, CruiseA (Diamond Princess cruise docked in Oakland, CA), USA, and Europe. The outer track shows the gene/protein coding regions displayed in blue. The four inner tracks display nucleotide frequencies (green (A), orange (C), blue (G), and red (T)) at 44 locations with an alternate allele frequency > 1%. The inner-most track of arcs shows variant locations having a high linkage with r^2^ values ranging from 0.8 (narrow, light) to 1 (wide, dark). Abbreviations: leader protein (LP), 3C-like-proteinase (3C-P), RNA-dependent-RNA-polymerase (RNA-Poly), 3'-to-5'-exonuclease (3’-5’-Exo), 2'-O-ribose-methyltransferase (2’ORM).

**Fig 10:**
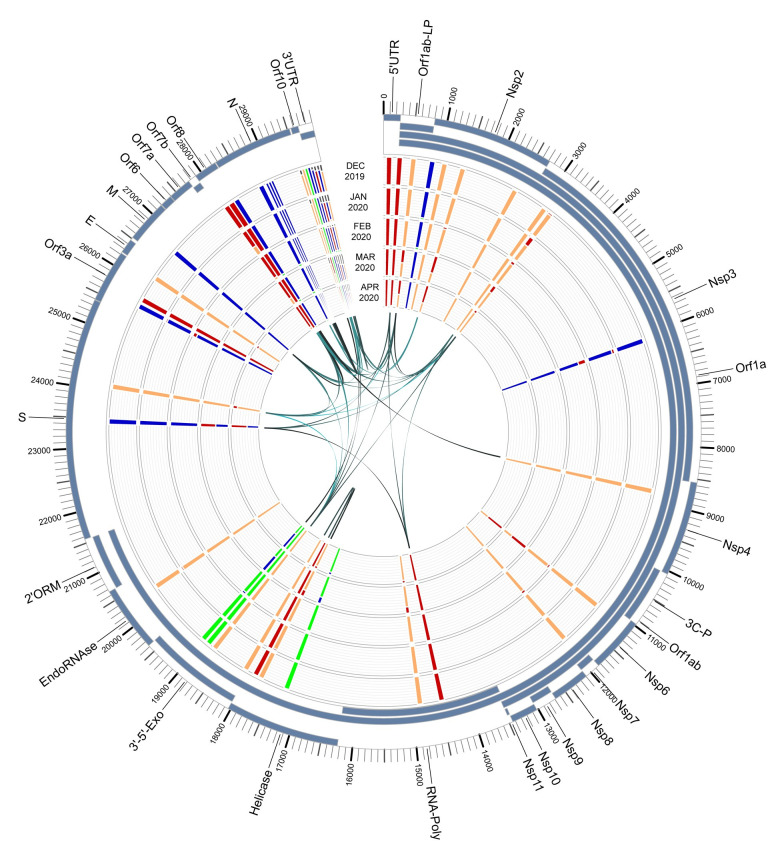
SARS-CoV-2 genome variants across time. Shown are the variants based on sampling date, ranging from December, 2019 to April, 2020. The outer track shows the gene/protein coding regions displayed in blue. The four inner tracks display nucleotide frequencies (green (A), orange (C), blue (G), and red (T)) at 44 locations with an alternate allele frequency > 1%. The inner-most track of arcs shows variant locations having a high linkage with r^2^ values ranging from 0.8 (narrow, light) to 1 (wide, dark). Abbreviations: leader protein (LP), 3C-like-proteinase (3C-P), RNA-dependent-RNA-polymerase (RNA-Poly), 3'-to-5'-exonuclease (3’-5’-Exo), 2'-O-ribose-methyltransferase (2’ORM).

To understand if the variant alleles were linked to each other, we analyzed the linkage between the detected variants using Haploview (v4.2) [24] (Figs 5 and 6). A total of 38 associations with an r^2^ value > = 0.8 were detected (Table 5). In order to further examine these associations for viral evolution, we looked at their frequencies within geographic groupings, including China, East Asia, Hong Kong and Taiwan (Fig 7); China, India, and West Asia (Fig 8); and China, CruiseA, USA, and Europe (Fig 9), as well as by sampling date (Fig 10). Intriguingly, the time analysis shows nine variants that are shifting frequency in samples more recently procured during March-April 2020, including 490T> A, 1397G> A, 23403A> G, and 29540G> A. Eight of these variants are in coding regions, with six producing non-synonymous mutations in the amino acid sequence. One of these, 23403A> G has been recently reported as a mutation in the Spike protein resulting in a more transmissible form of SARS-CoV-2 [19].

**Table 5:** Associations found with r^2^ > 0.8 in Haploview.

Locations	r^2^	Locations	r^2^
490/3177	1	3177/26729	0.927
8782/28144	1	3177/28077	0.927
14408/23403	1	490/29700	0.923
17747/17858	1	3177/29700	0.923
18998/29540	1	3037/14408	0.916
26729/28077	1	3037/23403	0.915
28882/28883	1	241/3037	0.912
241/14408	0.997	24034/26729	0.901
241/23403	0.997	24034/28077	0.901
28881/28882	0.981	18736/26729	0.891
28881/28883	0.981	18736/28077	0.891
29867/29868	0.972	1397/28688	0.874
17858/18060	0.968	1397/29742	0.874
17747/18060	0.967	28688/29742	0.861
490/18736	0.961	26729/29700	0.855
3177/18736	0.961	28077/29700	0.855
18736/29700	0.961	490/24034	0.835
490/26729	0.927	3177/24034	0.835
490/28077	0.927	18736/24034	0.802

## Discussion

Of the 44 common variants we found, 20 result in nonsynonymous mutations which may have functional relevance. Understanding these specific variants is critical to being able to react to the evolution of SARS-CoV-2, in particular, in developing an effective vaccine. Among previously reported variants, a few proposed functional consequences have been reported. 14408C > T within the RNA-dependent RNA polymerase gene (RdRp) may potentially affect proofreading or binding with other cofactors, thus affecting viral mutation rates [10]. The variant 23403G > A within the S protein has been hypothesized to increase transmissibility due to its expansion in global samples. This is thought to occur via one of two mechanisms, the first by diminishing interactions between the S1 and S2 promoters of the spike protein based on structural changes, and the second by affecting immunological response due to its location within an immune-dominant epitope [19].

Our analysis uncovered six previously unreported nonsynonymous variants, including 833T > C, 1059C > T, 11916 C > T, 18736T > C, 18998C > T, and 25563G > T. Five of these transition events occur within ORF1ab, and one occurs within ORF3a. Of these, two (833T > C and 1059C > T) are within the nsp2 region, which is postulated to play a role in the host cell survival pathway via interactions with prohibitin (PHB) and prohbitin 2 (PHB2); one (11916 C> T) is within the nsp7 region that may act as a primase and therefore be involved in viral replication; two (18376T > C and 18998C > T) are within the 3’ to 5’ exonuclease, which functions in proofreading; and one (25563G > T) in ORF3a which forms viroporin ion channels and may modulate virus release [25].

While the SARS-CoV-2 virus is the product of a single-stranded RNA genome and our analysis of variants having a high association may be more applicable to eukaryotic genomes, it is known that recombination between strains is a key contributor to coronavirus evolution [26–28]. Korber, et. al [19] demonstrate that one particular mutation in the S protein (S943P) is likely a result of recombination between strains, due to the fact that at the time of reporting, it was found only in Belgium, but in multiple lineages, suggesting it was not a result of a founder sequence. Many associations have been identified [10, 12, 15, 19], including the 38 we found with r^2^ > 0.8. Further analysis of these is necessary, in order to rule out founder effect. A follow-up study using the reverse-genetic approach would be needed to understand the biological effects of the variant nucleotides and the nature of the high association between the variant sequences (e.g. compensatory mutations) [29, 30].

## Conclusion

Our analysis resulted in 44 common variants within SARS-CoV-2, 24 of which had not been previously described. From these variants, a total of 38 pairwise associations had an r^2^ value > 0.8, indicating a high correlation. Analysis over time shows a shifting frequency of nine variants. One of these, 23403A > G, causes the D614G amino acid change in the spike protein. Recent studies have shown an increase of D614G genotypes in viral isolates (from 10% on March 1, 2020 to 78% on May 29, 2020), which is associated with lower viral loads [31]. Since COVID-19 is an emerging disease, a lot of current research efforts are focused on understanding the origin and mutation of the SARS-CoV-2 virus and isolates. As a result, the amount of sequence information is increasing on a daily basis. In this study, we constructed a framework that will allow us to update our analysis rather efficiently with little intervention. We hope to expand our analysis to include more recent submissions to the NCBI GenBank database, as well as the GISAID EpiFlu database [32]. As more data becomes available, with additional isolates resulting from community-acquired transmission, a more complete picture of the evolution of SARS-CoV-2 will be possible.

## Supporting information

S1 Table ISO 366–1 three letter country codes. (DOCX)

S2 Table Mutations previously identified. “X” represents allele found in isolate genome while “*” represents an alternate allele detected using deep sequencing.
(DOCX)
