# Supplementary material for: Variant analysis of 1,040 SARS-CoV-2 genomes
Source: PLoS One. 2020 Nov 5;15(11):e0241535. doi: 10.1371/journal.pone.0241535 (PMC7643988; doi:10.1371/journal.pone.0241535)
Supplement: S1 Table — (DOCX) [file pone.0241535.s001.docx]

**S1 Table.** **ISO 366-1 three letter country codes.**

| **Code** | **Country** |  | **Code** | **Country** |
| --- | --- | --- | --- | --- |
| AUS | Australia |  | KOR | South Korea |
| BRA | Brazil |  | LKA | Sri Lanka |
| CHN | China |  | MYS | Malaysia |
| CZE | Czech Republic |  | NPL | Nepal |
| DEU | Germany |  | PAK | Pakistan |
| ESP | Spain |  | PER | Peru |
| FRA | France |  | PRI | Puerto Rico |
| GRC | Greece |  | SWE | Sweden |
| HKG | Hong Kong |  | TUR | Turkey |
| IND | India |  | TWN | Taiwan |
| IRN | Iran |  | USA | United States |
| ISR | Israel |  | VNM | Vietnam |
| ITA | Italy |  | ZAF | South Africa |
| KAZ | Kazakhstan |  |  |  |
