# Supplementary material for: Variant analysis of 1,040 SARS-CoV-2 genomes
Source: PLoS One. 2020 Nov 5;15(11):e0241535. doi: 10.1371/journal.pone.0241535 (PMC7643988; doi:10.1371/journal.pone.0241535)
Supplement: S2 Table — “X” represents allele found in isolate genome while “*” represents an alternate allele detected using deep sequencing. (DOCX) [file pone.0241535.s002.docx]

**S2 Table.** **Mutations previously identified.** “X” represents allele found in isolate genome while “*” represents an alternate allele detected using deep sequencing.

| Gene | Study  Reference  Cutoff  N | Wang  [16]  UNK  108 | Tang  [15]  1  103 | Pachetti  [10] | Khailany  [14] | Wang  [13]  3  95 | Forster  [17] | Yin  [12]  UNK  558 | Shen  [18]  5%  8 |
| --- | --- | --- | --- | --- | --- | --- | --- | --- | --- |
| 5’UTR | 4A > T |  |  |  | X |  |  |  |  |
| 5’UTR | 16C > T |  |  |  | X |  |  |  |  |
| 5’UTR | 75C > A |  |  |  | X |  |  |  |  |
| 5’UTR | 104T > A |  |  |  | X |  |  |  |  |
| 5’UTR | 111T > C |  |  |  | X |  |  |  |  |
| 5’UTR | 112T > G |  |  |  | X |  |  |  |  |
| 5’UTR | 119C > G |  |  |  | X |  |  |  |  |
| 5’UTR | 120T > C |  |  |  | X |  |  |  |  |
| 5’UTR | 124G > A |  |  |  | X |  |  |  |  |
| 5’UTR | 241C > T |  |  |  | X |  |  | X |  |
| ORF1ab | 359-382del |  |  |  | X |  |  |  |  |
| ORF1ab | 376G > T |  |  |  |  |  |  |  | * |
| ORF1ab | 394T > C |  |  |  |  |  |  |  | * |
| ORF1ab | 490T > A |  | X |  | X |  |  |  |  |
| ORF1ab | 565T > C |  | * |  |  |  |  |  |  |
| ORF1ab | 614 |  | X |  |  |  |  |  |  |
| ORF1ab | 1397 |  |  | X |  |  |  |  |  |
| ORF1ab | 1440G > A |  |  |  |  |  |  |  | * |
| ORF1ab | 1497G > A |  |  |  | X |  |  |  |  |
| ORF1ab | 1548G > A |  |  |  | X |  |  |  |  |
| ORF1ab | 1623T > C |  |  |  |  |  |  |  | * |
| ORF1ab | 1636G > C |  |  |  |  |  |  |  | * |
| ORF1ab | 1821G > A |  | * |  |  |  |  |  | * |
| ORF1ab | 1912C > T |  |  |  | X |  |  |  |  |
| ORF1ab | 1914G > A |  |  |  |  |  |  |  | * |
| ORF1ab | 1991T > G |  |  |  |  |  |  |  | * |
| ORF1ab | 2297T > C |  |  |  |  |  |  |  | * |
| ORF1ab | 2662C > T |  | X |  | X | X |  |  |  |
| ORF1ab | 2721C > T |  |  |  |  |  |  |  | * |
| ORF1ab | 2891 |  |  | X |  |  |  |  |  |
| ORF1ab | 3036 |  |  | X |  |  |  |  |  |
| ORF1ab | 3037C > T | X |  |  |  |  |  | X |  |
| ORF1ab | 3177C > T |  | X |  | X |  |  |  |  |
| ORF1ab | 3683C > T |  |  |  |  |  |  |  | * |
| ORF1ab | 3778A > G |  |  |  | X |  |  |  |  |
| ORF1ab | 3792C > T |  |  |  | X |  |  |  |  |
| ORF1ab | 4122T > A |  |  |  |  |  |  |  | * |
| ORF1ab | 4771T > A |  |  |  |  |  |  |  | * |
| ORF1ab | 4794C > T |  |  |  |  |  |  |  | * |
| ORF1ab | 4795C > T |  | * |  |  |  |  |  |  |
| ORF1ab | 5084 |  | X |  |  |  |  |  |  |
| ORF1ab | 5214T > C |  |  |  |  |  |  |  | * |
| ORF1ab | 5869C > T |  |  |  |  |  |  |  | * |
| ORF1ab | 6027C > T |  |  |  |  |  |  |  | * |
| ORF1ab | 6031C > T |  |  |  | X |  |  |  |  |
| ORF1ab | 6035A > G |  |  |  | X |  |  |  |  |
| ORF1ab | 6051A > T |  |  |  |  |  |  |  | * |
| ORF1ab | 6254G > T |  |  |  |  |  |  |  | * |
| ORF1ab | 6360A > G |  | * |  |  |  |  |  |  |
| ORF1ab | 6492T > C |  |  |  |  |  |  |  | * |
| ORF1ab | 6496T > C |  |  |  |  |  |  |  | * |
| ORF1ab | 6503G > T |  |  |  |  |  |  |  | * |
| ORF1ab | 6766T > C |  |  |  |  |  |  |  | * |
| ORF1ab | 6968C > A |  |  |  | X |  |  |  |  |
| ORF1ab | 6996T > C |  |  |  | X |  |  |  |  |
| ORF1ab | 7016G > A |  |  |  | X |  |  |  |  |
| ORF1ab | 7042G > A |  | * |  |  |  |  |  |  |
| ORF1ab | 7866G > T |  |  |  | X |  |  |  | * |
| ORF1ab | 8001A > C |  |  |  | X |  |  |  |  |
| ORF1ab | 8242T > C |  |  |  |  |  |  |  | * |
| ORF1ab | 8286T > C |  |  |  |  |  |  |  | * |
| ORF1ab | 8615G > A |  |  |  |  |  |  |  | * |
| ORF1ab | 8388A > G |  |  |  | X |  |  |  |  |
| ORF1ab | 8615G > A |  |  |  |  |  |  |  | * |
| ORF1ab | 8782C > T | X | X | X | X | X | X | X |  |
| ORF1ab | 8987T > A |  |  |  | X |  |  |  |  |
| ORF1ab | 9064TTAT > TT |  | * |  |  |  |  |  |  |
| ORF1ab | 9534C > T |  |  |  | X |  |  |  |  |
| ORF1ab | 9805G > A |  |  |  |  |  |  |  | * |
| ORF1ab | 9989A > G |  |  |  |  |  |  |  | * |
| ORF1ab | 10024A > T |  |  |  |  |  |  |  | * |
| ORF1ab | 10481G > A |  |  |  |  |  |  |  | * |
| ORF1ab | 10625G > C |  |  |  |  |  |  |  | * |
| ORF1ab | 10771T > C |  |  |  |  |  |  |  | * |
| ORF1ab | 10779T > A |  |  |  |  |  |  |  | * |
| ORF1ab | 10817G > C |  |  |  |  |  |  |  | * |
| ORF1ab | 11083G > T | X | X | X | X | X |  | X |  |
| ORF1ab | 11147T > C |  |  |  |  |  |  |  | * |
| ORF1ab | 11148T > C |  |  |  |  |  |  |  | * |
| ORF1ab | 11367A > T |  |  |  |  |  |  |  | * |
| ORF1ab | 11557G > T |  |  |  | X |  |  |  |  |
| ORF1ab | 11563C > T |  | * |  |  |  |  |  |  |
| ORF1ab | 11749T > A |  |  |  | X |  |  |  |  |
| ORF1ab | 11764T > A |  |  |  | X |  |  |  |  |
| ORF1ab | 12153C > T |  | * |  |  |  |  |  |  |
| ORF1ab | 12258A > G |  |  |  |  |  |  |  | * |
| ORF1ab | 13225C > G |  |  |  | X |  |  |  |  |
| ORF1ab | 13226T > C |  |  |  | X |  |  |  |  |
| ORF1ab | 13899T > C |  |  |  |  |  |  |  | * |
| ORF1ab | 14408T > C |  |  | X |  |  |  | X |  |
| ORF1ab | 14438T > C |  |  |  |  |  |  |  | * |
| ORF1ab | 15324C > T |  | X |  | X |  |  |  |  |
| ORF1ab | 15597T > C |  |  |  | X |  |  |  |  |
| ORF1ab | 15921G > T |  | * |  |  |  |  |  |  |
| ORF1ab | 16080C > T |  |  |  |  |  |  |  | * |
| ORF1ab | 16474A > G |  | * |  |  |  |  |  |  |
| ORF1ab | 16467A > G |  |  |  | X |  |  |  |  |
| ORF1ab | 17190T > C |  |  |  |  |  |  |  | * |
| ORF1ab | 17373 |  | X |  |  | X |  |  |  |
| ORF1ab | 17423A > G |  |  |  | X |  |  |  |  |
| ORF1ab | 17746 |  |  | X |  |  |  |  |  |
| ORF1ab | 17747C > T |  |  |  |  |  |  | X |  |
| ORF1ab | 17825C > T |  | * |  |  |  |  |  |  |
| ORF1ab | 17857 |  |  | X |  |  |  |  |  |
| ORF1ab | 17858A > G |  |  |  |  |  |  | X |  |
| ORF1ab | 17934C > A |  |  |  |  |  |  |  | * |
| ORF1ab | 18060C > T |  | X | X | X | X |  | X |  |
| ORF1ab | 18488 |  | X |  |  |  |  |  |  |
| ORF1ab | 18512C > T |  |  |  | X |  |  |  |  |
| ORF1ab | 19164C > T |  | * |  |  |  |  |  |  |
| ORF1ab | 19879G> T |  |  |  |  |  |  |  | * |
| ORF1ab | 19948A > C |  |  |  |  |  |  |  | * |
| ORF1ab | 20141C > G |  |  |  |  |  |  |  | * |
| ORF1ab | 20240G > A |  |  |  |  |  |  |  | * |
| ORF1ab | 20344C > T |  | * |  |  |  |  |  |  |
| ORF1ab | 20670 |  | X |  |  |  |  |  |  |
| ORF1ab | 20679G > A |  | X |  | X |  |  |  |  |
| ORF1ab | 20703C > T |  |  |  |  |  |  |  | * |
| ORF1ab | 20716A > T |  |  |  |  |  |  |  | * |
| ORF1ab | 20815T > C |  |  |  |  |  |  |  | * |
| ORF1ab | 21137A > G |  |  |  | X |  |  |  |  |
| ORF1ab | 21316G > A |  |  |  | X |  |  |  |  |
| ORF1ab | 21383A > C |  |  |  |  |  |  |  | * |
| ORF1ab | 21386insT |  |  |  | X |  |  |  |  |
| ORF1ab | 21386C > T |  |  |  | X |  |  |  |  |
| ORF1ab | 21388-21390insTT |  |  |  | X |  |  |  |  |
| S | 21707 |  | X |  |  | X |  |  |  |
| S | 22432C > T |  |  |  | X |  |  |  |  |
| S | 22661G > T | X | X |  |  |  |  |  |  |
| S | 23127C > T |  |  |  |  |  |  |  | * |
| S | 23185C > T |  |  |  | X |  |  |  |  |
| S | 23308T > A |  |  |  |  |  |  |  | * |
| S | 23403A > G | X |  | X |  |  |  | X |  |
| S | 23569 |  | X |  |  |  |  |  |  |
| S | 23585C> T |  |  |  |  |  |  |  | * |
| S | 23605 |  | X |  |  |  |  |  |  |
| S | 23725T > A |  |  |  |  |  |  |  | * |
| S | 23920A > G |  |  |  |  |  |  |  | * |
| S | 23923A > C |  |  |  |  |  |  |  | * |
| S | 23952T > G |  |  |  | X |  |  |  |  |
| S | 24034C > T |  | X |  | X | X |  |  |  |
| S | 24265A > G |  |  |  |  |  |  |  | * |
| S | 24292A > G |  |  |  | X |  |  |  |  |
| S | 24323A > C |  | * |  |  |  |  |  |  |
| S | 24325A > G |  | X |  | X |  |  |  |  |
| S | 24404C > T |  |  |  |  |  |  |  | * |
| S | 24428G > A |  |  |  |  |  |  |  | * |
| S | 24816G > C |  |  |  |  |  |  |  | * |
| ORF3a | 25399T > G |  |  |  |  |  |  |  | * |
| ORF3a | 25590A > G |  |  |  |  |  |  |  | * |
| ORF3a | 25810C > G |  |  |  | X |  |  |  |  |
| ORF3a | 26102T > A |  |  |  |  |  |  |  | * |
| ORF3a | 26143 |  |  | X |  |  |  |  |  |
| ORF3a | 26144G > T | X | X |  | X | X | X | X |  |
| E | 26314G > A |  | * |  |  |  |  |  |  |
| M | 26590T > C |  | * |  |  |  |  |  |  |
| M | 26729T > C |  | X |  | X | X |  |  |  |
| M | 27046C > T |  |  |  |  |  |  | X |  |
| ORF6 | 27347A > T |  |  |  |  |  |  |  | * |
| ORF7a | 27403A > T |  |  |  |  |  |  |  | * |
| ORF7a | 27493C > T |  | X |  | X |  |  |  | * |
| ORF7a/b | 27864C > A |  |  |  |  |  |  |  | * |
| ORF8 | 27966T > C |  |  |  |  |  |  |  | * |
| ORF8 | 27971T > C |  |  |  |  |  |  |  | * |
| ORF8 | 28038G > A |  |  |  |  |  |  |  | * |
| ORF8 | 28077G > C |  | X |  | X | X |  |  |  |
| ORF8 | 28144T > C | X | X | X | X | X | X |  |  |
| ORF8 | 28253C > T |  | X |  | X |  |  |  | * |
| N | 28350G > A |  |  |  |  |  |  |  | * |
| N | 28805G > A |  |  |  |  |  |  |  | * |
| N | 28854C > T |  | X |  | X | X |  |  |  |
| N | 28878 |  | X |  |  |  |  |  |  |
| N | 28792A > C |  |  |  | X |  |  |  |  |
| N | 28881G > A |  |  | X |  |  |  | X |  |
| N | 28882G > A |  |  |  |  |  |  | X |  |
| N | 28883G > C |  |  |  |  |  |  | X |  |
| N | 29039A > T |  |  |  |  |  |  |  | * |
| N | 29095C > T |  | X |  | X | X | X |  |  |
| N | 29140G > C |  |  |  |  |  |  |  | * |
| N | 29212G > C |  |  |  |  |  |  |  | * |
| N | 29303C > T |  | X |  | X |  |  |  |  |
| N | 29398G > C |  |  |  |  |  |  |  | * |
| N | 29441C > A |  | * |  |  |  |  |  |  |
| N | 29513G > A |  |  |  |  |  |  |  | * |
| N | 29515T > A |  |  |  |  |  |  |  | * |
| N | 29516G > A |  |  |  |  |  |  |  | * |
| ORF10 | 29642C > T |  |  |  | X |  |  |  |  |
| 3’UTR | 29705G > T |  |  |  | X |  |  |  |  |
| 3’UTR | 29749-29759del |  |  |  | X |  |  |  |  |
| 3’UTR | 29854C > T |  |  |  | X |  |  |  |  |
| 3’UTR | 29856T > A |  |  |  | X |  |  |  |  |
| 3’UTR | 29869del |  |  |  | X |  |  |  |  |
